# Current Status, Recent Advances, and Main Challenges on Table Olive Fermentation: The Present Meets the Future

**DOI:** 10.3389/fmicb.2021.797295

**Published:** 2022-01-13

**Authors:** Dimitrios A. Anagnostopoulos, Dimitrios Tsaltas

**Affiliations:** Department of Agricultural Sciences, Biotechnology and Food Science, Cyprus University of Technology, Limassol, Cyprus

**Keywords:** table olives, fermentation, microbiota, starter cultures, functional table olives, high throughput sequencing, multi-omics, microbial terroir

## Abstract

Table olives are among the most well-known fermented foods, being a vital part of the Mediterranean pyramid diet. They constitute a noteworthy economic factor for the producing countries since both their production and consumption are exponentially increasing year by year, worldwide. Despite its significance, olive’s processing is still craft based, not changed since antiquity, leading to the production of an unstable final product with potential risk concerns, especially related to deterioration. However, based on industrial needs and market demands for reproducible, safe, and healthy products, the modernization of olive fermentation processing is the most important challenge of the current decade. In this sense, the reduction of sodium content and more importantly the use of suitable starter cultures, exhibiting both technological and potential probiotic features, to drive the process may extremely contribute to this need. Prior, to achieve in this effort, the full understanding of table olive microbial ecology during fermentation, including an in-depth determination of microbiota presence and/or dominance and its functionality (genes responsible for metabolite production) that shape the sensorial characteristics of the final product, is a pre-requisite. The advent of meta-omics technology could provide a thorough study of this complex ecosystem, opening in parallel new insights in the field, such as the concept of microbial terroir. Herein, we provide an updated overview in the field of olive fermentation, pointing out some important challenges/perspectives that could be the key to the olive sector’s advancement and modernization.

## Introduction

Table olives are among the most popular and healthy fermented fruits and vegetables, with a great socio-economic impact, worldwide. They have been cultivated along the Mediterranean basin for more than 5,000 years. According to the International Olive Oil Council (IOOC) (2004), table olive is “the product prepared from the sound fruits of varieties of the cultivated olive trees (*Olea europaea L.*) that are chosen for their production of olives whose volume, shape, flesh-to-stone ratio, fine flesh, taste, firmness, and ease of detachment from the stone make them particularly suitable for processing; treated to remove their bitterness and preserved by natural fermentation; or by heat treatment, with or without the addition of preservatives; packed with or without covering liquid.” Nowadays, olive has been recognized as more than just another fermented product (Perpetuini et al., 2020), while it is considered as the food of the future (Bonatsou et al., 2017). Last season, the global production reached close to 3,000,000 tons and is increasing exponentially year to year, with the top producer being Spain, followed by Italy and, to a lesser extent, other Mediterranean countries (e.g., Greece, Algeria, Turkey, Egypt, and Portugal) [International Olive Oil Council (IOOC), 2020b]. However, the production beyond the Mediterranean area (e.g., South America, Middle East, and Australia) is also noteworthy [International Olive Oil Council (IOOC), 2020b]. The worldwide consumption is also increasing year to year, amounting to more than 2,000,000 tons/year, with Egypt, the United States, Turkey, and Algeria as the top consumers [International Olive Oil Council (IOOC), 2020a].

Despite its significant impact especially on the producing countries, the processing of olive is still empirical, leading to the production of an unstable and non-reproducible final product, with potential safety concerns (Heperkan, 2013). In the last two decades, a huge number of scientific findings supported the application of starter cultures at the beginning of olive’s processing to drive the fermentation, in some cases, even a lower sodium content, in order to produce a stable, reproducible, and healthier product (Panagou et al., 2008; Papadelli et al., 2015; Pino et al., 2018, 2019; Anagnostopoulos et al., 2020a,b; Benítez-Cabello et al., 2020a). However, the use of starters at an industrial-scale olive fermentation is yet to be established, even though scientific findings still support and enhance this concept, until nowadays.

Table olives are categorized among the most health-promoting products since they include a variety of bioactive compounds with potential functional properties. For instance, they contain organic acids, vitamins, trace elements, and a polyphenol profile with important antimicrobial and antioxidant capacities (Botta and Cocolin, 2012). The latter is mainly attributed to the presence of the secoroides molecule of oleuropein, which is responsible for raw olive bitterness (Heperkan, 2013). During fermentation, oleuropein is hydrolyzed *via* the microbial enzymatic activity of β-glucosidase, releasing other simpler molecules (glucose and oleuropein aglycone). Afterward, those derivatives are further degraded by esterase activity in other non-bitter compounds, such as hydroxytyrosol and elenolic acid, which are also exhibiting high antimicrobial and antioxidant activities (Bianchi, 2003), making in parallel the final product suitable for consumption. The ratio of those molecules is strongly linked with the olive’s microbiota profile, which, in turn, is influenced by other factors, such as the type of fermentation. According to the International Olive Oil Council (IOOC) (2004), there are four main practices, all of them spontaneous, to produce table olives: (1) the Spanish style, in which raw olives are subjected to lye treatment (NaOH ∼1.8–2.5% *w*/*v*) for debittering, prior to brining; (2) the natural or Greek style, where olives are directly brined (NaCl ∼8–10% *w*/*v*) without any pre-treatment; (3) the Californian style, in which olives are darkened by oxidation; and (4) other types based on local practices (Perpetuini et al., 2020). Furthermore, NaCl content and the temperature during processing are also playing a key role in microbiota formation during processing (Tassou et al., 2002; Arroyo-López et al., 2008a). Finally, the different varieties, as well as the origin of olives, may also shape the dominant microbiota that drives the whole process. As a consequence, all the aforementioned factors are strongly affecting, directly or indirectly, the organoleptic characteristics of the final product (Bonatsou et al., 2017).

Thus, it is clearly observed that all the aforementioned parameters must be deeply studied, in order to fully understand the fraction that influences the microbial consortium in this complex ecosystem. This will lead to strengthening the effort of standardization of the fermentation process, which is attempted especially in the last two decades. In this sense, the advent of high-throughput sequencing (HTS) technology has revolutionized the research in food microbiology (De Filippis et al., 2017). This set of methods represents a modernized way on how scientists perceive and verge the microbial ecology and its complexity in an ecosystem (Cocolin et al., 2018), opening new insights in better understanding the microbial ecology of several fermented foods. Nevertheless, the up-to-date use of such methods in the field of olive fermentation is still in its infancy.

Moreover, in the last years, several studies support the claim of microbial-based geographical fingerprint in several fermented products, such as wine (Bokulich et al., 2014) and meat products (Van Reckem et al., 2019), noting that different environments are hosting, beyond the cosmopolitan, specific/unique microbial fractions. In the field of table olives, this concept was officially started to be studied by the end of the last decade (Lucena-Padrós and Ruiz-Barba, 2019) and undoubtedly deserves further attention in the near future.

The present article provides an updated overview regarding the up-to-date knowledge on microbial communities present during olive processing, as well as the technological advances applied, pointing out some important challenges and perspectives that both industrial and scientific communities will face in the coming years.

## Table Olive Fermentation Microbiota

As previously mentioned, since the fermentation of table olive is an entirely spontaneous process, the formed microbiota that drives the whole process is coming from the olive drupe and its micro-environment (Bonatsou et al., 2017), which is strongly linked with cultivar, year, and the type of fermentation, which, in most cases, is based on local tradition (Perpetuini et al., 2020). In general, lactic acid bacteria (LAB) and yeasts constitute the dominant microbiota from the middle of the fermentation and thereafter, whereas other microorganisms such as Enterobacteriaceae, *Clostridium*, *Pseudomonas*, *Staphylococcus*, *Vibrio*, and molds are in high abundance in raw olives, as well as at the first period of fermentation (Panagou et al., 2003; Randazzo et al., 2012; Lucena-Padrós et al., 2015; Papadelli et al., 2015; Bavaro et al., 2017; Bonatsou et al., 2018b; Pino et al., 2018, 2019; Posada-Izquierdo et al., 2021). The role of LAB is to rapidly convert sugars into organic acids to decrease brine’s pH and thus stabilize the fermentation process by inhibiting the undesirable microbial groups mentioned above, since they are unable to grow in acidic conditions (Heperkan, 2013). On the other hand, although yeasts were supposed to be an undesirable microbial group for many years, this consideration was totally revised in the last two decades. According to the literature, yeasts are contributing to crucial sensorial attribute improvement such as taste, odor, and flavor, mainly *via* their enzymatic activity, as well as their capacity to produce several metabolites, especially volatile organic compounds (VOCs) (Bonatsou et al., 2017). Furthermore, the invaluable contribution of yeasts in the enhancement of LAB growth has also been noted (Arroyo-López et al., 2012a,b). The latter is achieved *via* the capacity of yeasts (1) to degrade several phenolic compounds, which act as inhibitors for LAB growth, and/or (2) to produce several compounds, which are crucial for LAB growth (vitamins, purines, etc.) (Arroyo-López et al., 2012a; Anagnostopoulos et al., 2017). Thus, nowadays, yeasts are considered an essential part of microbial consortia for succeeding in appropriate olive fermentation.

According to the literature, the ratio between LAB and yeasts and which of them would dominate during olive processing are strongly dependent on the type of processing (Perpetuini et al., 2020). More specifically, in Spanish-style processing, LAB are usually dominant and lead the fermentation (Doulgeraki et al., 2013; Lucena-Padrós et al., 2014). On the other hand, in natural style, despite that LAB are present, their dominance is influenced by several factors such as sugar availability, as well as the concentration of phenolic compounds that are released from drupe to brine and may inhibit their population. In this case, yeasts may become the predominant microbial group and thus are responsible for the course of the process and the formation of the sensorial attributes of the final product. The dominance of yeasts leads to the production of olives with specific characteristics, such as milder taste and less shelf life (Panagou et al., 2008). However, when yeasts and LAB coexist, the outcome is mainly reflected to taste, flavor, and texture improvement and thus better acceptance by consumers (Arroyo-López et al., 2008b; Lanza, 2013; Bleve et al., 2015).

Among LAB, the most abundant genera are former-*Lactobacillus* (thereafter *Lactiplantibacillus*), *Pediococcus*, *Leuconostoc*, and *Enterococcus*, while *Lactiplantibacillus plantarum*, *Lactiplantibacillus pentosus*, *Lacticaseibacillus casei*, and *Leuconostoc mesenteroides* are the most detected species (Randazzo et al., 2004; Panagou et al., 2008; Bleve et al., 2014; Pino et al., 2018). Regarding yeasts, several genera such as *Candida* (*C. diddensiae*, *C. boidinii*, and *C. tropicalis*), *Pichia* (*P. membranifaciens*), *Saccharomyces* (*S. cerevisiae*), *Debaryomyces* (*D. hansenii*), and *Wyckerhamomyces* (*W. anomalus*) have been detected as the main part of the dominant microbiota (Tofalo et al., 2013; Pereira et al., 2015; Bonatsou et al., 2018b). Furthermore, the presence of several genera of molds (e.g., *Penicillium* and *Aerobasidium*) isolated mainly from fermented black table olives (Cellina di Nardò, Leccino, Kalamàta, and Conservolea) has been recently noted (Bavaro et al., 2017; Bonatsou et al., 2018b).

As previously mentioned, the presence of several members belonging to Enterobacteriaceae, *Pseudomonas*, *Vibrio*, and *Clostridium* was noted in raw olives and/or at the beginning of fermentation. In a successful fermentation, these microbial groups should be eliminated in the early stages and should not be detected in the middle and especially at the end of processing. For instance, according to Alves et al. (2012), the presence of Enterobacteriaceae was noteworthy at the beginning of processing. However, at the final stage of fermentation, their population was below the detection limit. Similar findings were also highlighted by other studies (Papadelli et al., 2015; Pino et al., 2018, 2019; Anagnostopoulos et al., 2020a,b).

However, spontaneous fermentation is not fully predictable and has several disadvantages, such as a potential growth of undesirable microbes at an advanced level of fermentation, which, in turn, could provoke fermentation abnormality, *via* a plethora of metabolic products, resulting in the production of unsuitable and/or unacceptable final product (Bonatsou et al., 2018a). The presence of such microbes is the result of inadequate and/or not rapidly reducing pH, which is linked with low metabolism of LAB, when their population does not exhibit high levels. Indeed, Lanza (2013) noted the potential growth of *Clostridium* spp. at high brines’ pH, resulting in several off flavors and off odors, *via* the production of undesired metabolites (e.g., butyric acid). Pereira et al. (2008) detected *Clostridium* spp. in almost all examined table olives commercialized in Portugal, which was attributed to high pH in both pulps and brines, as well as to the resistance of *Clostridium* spores to pasteurization. Additionally, in such circumstances, the growth of *Propionibacterium* can be favored. This microbe is able to provoke the so-called “zapatera,” which is a phenomenon responsible for the formation of cyclohexane carboxylic acid (Tufariello et al., 2015), leading to malodors, as well as a potential production of undesired biogenic amines (Perpetuini et al., 2020).

## Technological Advancements on Olive Fermentation Processing

### Reduction/Replacement of Sodium Chloride

Last decade, the reduction of sodium chloride levels used in olive processing has been strongly recommended, since according to the World Health Organization (WHO) (2007), the high amounts of salt intake are responsible for several health concerns, such as hypertension and cardiovascular diseases. Since table olives are subjected to brines with high NaCl content (8–10% or even higher), they do not harmonize to the global guidelines and suggestions, where the highest sodium intake has been recommended not to exceed 5 g per day. Thus, the reduction of sodium content during olive fermentation has gained the attention of both the scientific and industrial communities (Bautista-Gallego et al., 2013b), being one of the main challenges of the last decade. However, the total and/or partial removal of sodium content carries several risk concerns regarding the fermentation course, as well as the final product. More specifically, this strategy may favor the growth of undesirable microorganisms (pathogens and/or spoilage), leading not only to major discards due to safety aspects but also to product rejection by the consumers and thus major economic losses for the industry. Considering this, many attempts have been applied to produce olives with reduced NaCl levels, using a mix of other chloride salts, such as KCl, CaCl_2_, and ZnCl_2_ (Panagou et al., 2011; Bautista-Gallego et al., 2015). It is crucial to mention that in some cases, table olives produced by partial substitution of NaCl did not exhibit any safety issue (Bautista-Gallego et al., 2010, 2011, 2013b; Mantzouridou et al., 2020). However, other potential impacts and/or side effects on the sensorial attributes of olives fermented with partial substitution of sodium is still understudied. Indeed, the potential risk of sensorial abnormality and off-flavor production has been underlined, through the use of such alternatives (Zinno et al., 2017). Furthermore, López-López et al. (2016) noted a bitter taste in olives fermented by partial substitution of NaCl (by CaCl_2_), indicating that this strategy may lead to the product’s consumer rejection. Even though the authors highlighted some interesting sensorial improvements, regarding olives’ texture and color, it would be an omission to forget that bitterness is the most important parameter taken into account by the consumers. Panagou et al. (2011) studied the potential impact of different mixtures (NaCl, KCl, and CaCl_2_) during fermentation of Conservolea black olives, indicating that the equal combination of NaCl and KCl (4% *w*/*v* each) resulted in an acceptable final product with interesting sensorial attributes, including low score in bitterness, as revealed by the organoleptic analysis. Finally, in a recent study by Lanza et al. (2020), the effects of iodized salt on the sensorial attributes of naturally fermented table olives (cv. Carolea and cv. Leucocarpa) were evaluated. Results did not show any significant differences regarding sensorial and color traits between the control and treated samples, indicating the need for similar works to confirm the potential use of such alternative salt as a promising substitution strategy.

NaCl substitution on olive fermentation is still one of the major challenges for the industry, constituting several difficulties, as previously mentioned, such as safety concerns and acceptance of the final product. Thus, other strategies have arisen to achieve NaCl reduction. The most promising one is the use of a starter culture to produce olives with reduced NaCl content, without any other salt replacement (Fadda et al., 2014). Indeed, Pino et al. (2018, 2019) used LAB starters to drive the fermentation process of Nocellara Etnea table olives. The findings indicated that the reduction of NaCl content to 5% (*w*/*v*) is feasible, since not only no deterioration was observed but also the final product was characterized as acceptable by the sensory panel. Similar findings were highlighted by Anagnostopoulos et al. (2020a,b), where both inoculated Cypriot and Picual table olives with reduced NaCl content (from 10 to 7% *w*/*v*) were highly appreciated by the judges, while the final products exhibited stability and safety.

### Replacement of NaOH in Spanish-Style Green Olives Debittering

Chemical debittering is a pre-treatment method applied exclusively in Spanish-style green olives, to shorten the time of olives’ debittering (and thus the whole processing) *via* the use of NaOH solution (∼1.5–3%), which leads to the hydrolysis of oleuropein molecule (Cocolin et al., 2013a). This is the most well-known practice worldwide. However, it is crucial to highlight the two sides of the same coin. From one side, it constitutes an important practice with an economic impact for the producers, since it contributes to olives’ production in a shorter period than those produced by direct brining. On the other hand, several drawbacks have been noted for this application, especially in the last years. For instance, the use of NaOH leads to the elimination of several attributes related to both technological (i.e., aroma, VOCs, and texture) and health (i.e., nutritional and molecules with functional potential) properties (Tufariello et al., 2016). Furthermore, the chemical treatment strongly affects the microbiota that will drive the fermentation process. Indeed, according to Cocolin et al. (2013a), in non-treated Nocellara Etnea olives, the dominance of halophilic bacteria and *Lactiplantibacillus* was profound, whereas treated olives were characterized by the presence of enterobacteria. This indicates a potential risk concern regarding the fermentation course, as well as the final product. Moreover, the environmental issue that this practice provokes, *via* the high amounts of wastewater, should be taken seriously under consideration (Tufariello et al., 2016). Finally, nowadays, the increasing interest toward the green development (and the production of related products), as well as the consumer’s demand for natural products, requires a strong awareness of the scientific and technological communities to provide solutions that harmonize both an eco-friendly character and sustainability (Bevilacqua et al., 2015), as well as enhance the concept of health-promoting foods.

Thus, the trends nowadays deal with the total replacement of NaOH in olives’ debittering by other methods. One of the most promising applications is the use of a starter culture exhibiting a strong β-glucosidase activity, to hydrolyze the molecule of oleuropein in a short time. This topic is described more comprehensively in section “The Concept of a Starter Culture.” Another alternative practice is the use of other compounds that, as industrial waste, could be re-used for other practices. For instance, the use of KOH has been proposed by García-Serrano et al. (2019), since the wastewater from olives’ KOH debittering could be used for other agronomy practices. The authors noted that the use of KOH in debittering of Manzanilla and Hojiblanca cultivars resulted in a typical lactic fermentation, with non-deteriorated sensorial characteristics, except from a bit softer texture. However, it is crucial to highlight the high production of hydroxytyrosol, indicating a potential functionality. In another study, Habibi et al. (2016) introduced the application of power ultrasound as a promising method of olives’ debittering. The findings indicated a satisfactory removal rate of bitterness, decreasing in parallel the whole processing time to 1/3 (∼37%). All the aforementioned practices need further attention in the near future, since they potentially constitute the alternative way of olives’ debittering.

### The Concept of a Starter Culture

The use of starter cultures has been established in several fermented foods at an industrial scale since decades before, a fact that is yet to happen in the olives’ field. However, according to the literature, there is a huge amount of studies supporting this claim. More specifically, the use of starter cultures, either LAB (Panagou and Tassou, 2006; Papadelli et al., 2015; Pino et al., 2018, 2019; Anagnostopoulos et al., 2020a,b), yeasts (Ciafardini and Zullo, 2019; Tufariello et al., 2019), or a mix culture (LAB and yeast) (Benítez-Cabello et al., 2020a; Chytiri et al., 2020; Garrido-Fernández et al., 2021), has proven to safely drive and control the whole process, leading to the production of a stable and reproducible final product with enhanced organoleptic characteristics, as well as potential functional properties. The latter is mirrored by the work of Anagnostopoulos et al. (2020b), where brine’s inoculation with a *L. plantarum* starter led to the production of olives exhibiting high antioxidant capacity, as well as high levels of hydroxytyrosol, as a result of high oleuropein degradation activity exhibited by the LAB starter. Furthermore, Garrido-Fernández et al. (2021) reported a unique VOC profile production by using a mix culture of LAB (LPG1) and yeast (Y12) strains, such as phenylethyl acetate (attributed to sweet roses) and *cis*-2-penten-1-ol (responsible for green aroma tastes). Thus, brines’ inoculation is the key for a reproducible product of high quality with potential functionality and increased added value.

However, appropriate microbial starters should exhibit some specific biotechnological and safety traits, such as (a) easy and rapid adaptation to the brine’s environment (e.g., temperature, pH, and phenolic profile), (b) rapid domination against indigenous microbiota, (c) rapid brine pH reduction *via* the production of organic acids, (d) high enzymatic activity to contribute to the enhancement of sensory characteristics of the final product, and (e) the ability to rapidly degrade oleuropein molecule, *via* the enzymatic activity of β-glucosidase and esterase, making the product edible in a shortened period (Heperkan, 2013; Bonatsou et al., 2017). Finally, those microbes should be generally recognized as safe (GRAS), to ensure the safety of the product. According to the literature, there are several *in vitro* assays to evaluate the aforementioned technological properties. For instance, Hernández et al. (2007) examined the potential presence of some undesirable enzymatic capacities in several isolated yeast strains, such as pectinolytic and xylanolytic activities, which are responsible for texture softening, and thus, the positive strains were excluded from the selection of candidate starters. Abriouel et al. (2012) noted a satisfactory growth of several LAB strains at low temperature (10°C), as well as high acidification activity from the majority of them. Similarly, Iorizzo et al. (2016) revealed a very promising acidification capacity of all tested LAB strains, which also exhibited high tolerance to several pH ranges (6–8), salt concentrations, and temperatures (15–30°C). In the same study, all strains exhibited high survival in phenol (0.3% total phenol content), as well as high oleuropein degradation activity. Additionally, Vaccalluzzo et al. (2020a) studied the effects of stress parameters on the growth and oleuropein-degrading abilities of *L. plantarum* strains, indicating that low temperature (16°C) is the main factor that negatively affects both the growth as well as the oleuropein-degrading capacity of the studied strains. Bonatsou et al. (2018a) studied, besides other properties such as enzymatic activity, the tolerance of 49 yeast strains, isolated from Kalamata table olives, in several NaCl concentrations and pH values. Results indicated that most of them showed satisfactory tolerance to high salt content, while 27 of them showed β-glucosidase activity. Additionally, Bevilacqua et al. (2010) evaluated the survival of LAB strains at several pH, sodium contents, and temperatures, indicating very promising results for the majority of the studied strains.

Despite the large amount of research regarding the evaluation of technological features of indigenous LAB and yeasts, the *in vitro* assays are applied independently to each other, and thus, the findings may not reflect the real capacity of the tested microorganisms. To overcome this concern, the evaluation of technological properties in an environment that mimics the real conditions taking place during fermentation has been recommended and introduced by Bleve et al. (2014, 2015), who studied the tolerance of isolated LAB and yeasts strains in synthetic model brines. Those brines included several compounds that are usually generated during fermentation (oleuropein, hydroxytyrosol, etc.), and thus, they have been used as a reliable substrate to study the survival of the tested microbes. In this sense, similar studies are mandatory, while other assays in model brines (e.g., oleuropein degradation and/or acidification activity) should be applied in future works, to obtain useful and more reliable results (than those obtained by the conventional assays), in an attempt to select the most appropriate microbes to be used as starter cultures.

Except from the technological characteristics and based on consumers’ demand for healthier products, a starter culture should also exhibit other features, related to potential functional properties. This is an active scientific field with great biotechnological interest, which has arisen in the last two decades, to produce functional table olives, offering specific health benefits to the consumers. The concept of functional foods was firstly introduced in the 1980s, and their first definition was “foods for specified health use” (FOSHU). According to the Food and Agriculture Organization of the United Nations (FAO) (2007), a worldwide accepted definition is that functional foods are defined as “foods similar in appearance to conventional foods that are consumed as part of a normal diet and have demonstrated physiological benefits and/or the capacity to reduce the risk of chronic disease beyond their basic nutritional functions.” Among others, probiotics represent one of the most popular strategies to produce functional foods (Nagpal et al., 2012). According to the Food and Agriculture Organization of the United Nations/World Health Organization (FAO/WHO) (2002), “probiotics are live microorganisms which, when administered in adequate amounts, as part of a food or a supplement, confer some kind of health benefit on the host.” Usually, probiotics are microbes that are isolated from several human (Haller et al., 2001; Nueno-Palop and Narbad, 2011; Archer and Halami, 2015; Riaz Rajoka et al., 2017) or food sources, especially those subjected to natural fermentation (Haller et al., 2001; Marco et al., 2017; Sharma et al., 2018; Garcia-Gonzalez et al., 2021). It has been already noted that microbes should exhibit specific features, in order to be considered as potential probiotics. Among others, antimicrobial activity against pathogens and/or spoilers, antioxidant capacity, cholesterol-reduction activity, auto and co-aggregation capacities, survival during gastric and pancreatic digestion, and adhesion to epithelial cells are the most desirable attributes, with great scientific interest, while several *in vitro* assays have already been established to evaluate each of those characteristics (Bonatsou et al., 2017). However, it is crucial to point out that findings by those tests do not necessarily mean a real impact on human health. Thus, more advanced studies, such as clinical trials, should be applied to confirm or refuse the findings of the *in vitro* ones [Food and Agriculture Organization of the United Nations/World Health Organization (FAO/WHO), 2002]. Furthermore, the concept of bio-protective cultures is gaining more and more interest, due to the potential of those microorganisms to confer some kind of antimicrobial activity during food’s post-fermentation and storage period, leading to extension of a product’s shelf life without any risk and health concerns (Abouloifa et al., 2021). In the case of olives, Lavermicocca et al. (2018) inoculated a *L. plantarum* strain to ready-to-sell table olives. The survival of the strain was confirmed at high levels to both olives’ surface and brine, while the sensory score of the product was very high. They also applied a challenge test to *Listeria monocytogenes* inoculated in the brines. The results indicated that the *D*-value of the pathogen was reduced by 35 days in the bio-preserved product, while the preservation capacity was still active for almost 5 months. This kind of application provides a promising alternative way to chemical or thermal preservation treatments at post fermentation stage, and thus, scientists have to keep researching this interesting topic.

Up to date, the concept of probiotics is almost exclusively implicated in dairies. However, the need for alternative sources of probiotics has risen, mainly due to the appearance of new consumer categories (vegans, vegetarians, etc.), as well as the increasing lactose intolerance or allergic-to-milk-protein population (Kumar et al., 2015). Besides such food sources, several vegetables like table olives are the most promising ones, as they act as carriers for probiotics, due to their specific intrinsic structure (pores, lesions, lenticels, and irregularities), favoring probiotics’ colonization on their surface (Peres et al., 2012; Martins et al., 2013). The latter contributes to the protection of probiotics during digestion, ensuring their survival in adequate amounts (Bonatsou et al., 2017). Indeed, in a clinical study by Lavermicocca et al. (2005), several LAB strains belonging to the genera *Lactiplantibacillus* and *Bifidobacteria* were recovered from humans’ feces, in large populations, indicating that table olives have the potential to become a highly promising functional food.

For all the aforementioned, last decade, the interest of the scientific community was focused on the isolation of autochthonous microbes, to study beyond their technological characteristics described above, their potential probiotic properties. Indeed, there are several studies dealing with the probiotic characterization of LAB (Bevilacqua et al., 2010; Ghabbour et al., 2011; Abriouel et al., 2012; Argyri et al., 2013; Bautista-Gallego et al., 2013a; Bleve et al., 2014, 2015; Botta et al., 2014; Montoro et al., 2016; Anagnostopoulos et al., 2018; Guantario et al., 2018; Yalçınkaya and Kılıç, 2019; El Issaoui et al., 2021) and yeast/mold (Hernández et al., 2007; Bevilacqua et al., 2009, 2013; Silva et al., 2011; Bonatsou et al., 2015, 2018a; Bavaro et al., 2017; Oliveira et al., 2017; Porru et al., 2018; Mujdeci and Ozbas, 2020; Simões et al., 2021) strains, isolated from several olive varieties, in order to select and use those with the most promising features to produce table olives with potential health benefits. It is crucial to point out that in the majority of the studies, strain selection is based on the most combined range of both technological and probiotic features. It is noteworthy that in some previous studies, the significant contribution of principal component analysis (PCA), to discriminate the strains with the best combination features, was highlighted and recommended as the most appropriate statistical tool to be applied for such kind of studies (Rodríguez-Gómez et al., 2012; Bautista-Gallego et al., 2013a; Oliveira et al., 2017; Anagnostopoulos et al., 2018). As a representative example, Bautista-Gallego et al. (2013a) used PCA for the discrimination of 111 LAB isolates, which were examined for several potential properties (autoaggregation, hydrophobicity, bacteriocin production, etc.). The analysis highlighted a unique profile of four *L. pentosus* isolates that contained the best combination features. Those isolates were proposed as the most promising to be used as starters in olive fermentation.

Despite the continuing, from several studies, emergence of novel promising strains, isolated from several olive varieties or other fermented products, the application of starter cultures (either commercial or autochthonous) to drive olive fermentation at an industrial scale is still far from its official establishment (Campus et al., 2015; Tufariello et al., 2015; Bonatsou et al., 2017; Perpetuini et al., 2020). However, as already noted, several studies were applied, in the last two decades, using starter cultures to lead the fermentation process (Panagou and Tassou, 2006; Blana et al., 2014; De Angelis et al., 2015; Papadelli et al., 2015; Comunian et al., 2017; Randazzo et al., 2017; Chranioti et al., 2018; Pino et al., 2018, 2019; Anagnostopoulos et al., 2020a,b; Paba et al., 2020). As shown in Table 1, the majority of the studies support the use of starters to improve the quality and/or enhance the functional properties and/or improve the organoleptic characteristics of the final product. As a representative example, Chranioti et al. (2018) reported that the use of a *L. plantarum* starter shortened the fermentation time of green table olives, contributing in parallel to the improvement of the quality and the flavor of the final product. In another noteworthy study, Pino et al. (2019) proposed a sequential inoculation strategy (inoculation of a β-glucosidase-positive *L. plantarum* strain at the beginning of the process, followed by the inoculation of a *L. paracasei* probiotic strain after 60 days) to ferment Nocellara Etnea table olives. The authors reported a successful fermentation achieved in a short period, while the sequential inoculation in combination with reduced salt contents contributed to both polyphenol content increase, as well as the enhancement of the sensorial attributes of the final product. Similar findings were also highlighted by Anagnostopoulos et al. (2020a,b), where a commercial *L. plantarum* strain was used to drive the fermentation of both Cypriot and Picual olives. The authors highlighted the contribution of the starter in increasing the production of lactic acid, polyphenol, and hydroxytyrosol contents, resulting in both high antimicrobial and antioxidant capacities of the final products, indicating the key role of an appropriate starter for a successful fermentation process.

**TABLE 1:** Application of starter cultures in different fermentation types of several table olive cultivars.

Table olive	Fermentation type/conditions	Starter culture	Main findings^1^	References
Bella di Cerignola	Spanish style Brining conditions: 4 and 8% (*w*/*v*) NaCl Temperature: room temperature and 4°C	*L. paracasei* IMPC2.1	✓ Adhering on olive surface ✓ Higher pH reduction ✓ Production of a safe low-salt and probiotic product	De Bellis et al., 2010
Bella di Cerignola	Natural style Brining conditions: 7% (*w*/*v*) NaCl	Commercial *L. plantarum* Mix of commercial *L. plantarum* and autochthonous *W. anomalus* DiSSPA73 Mix of commercial *L. plantarum*, *W. anomalus* DiSSPA73, autochthonous *L. plantarum* DiSSPA1A7, and *L. pentosus* DiSSPA7	✓ Higher acidification ✓ Lower abundance of Enterobacteriaceae, *Propionibacterium*, and *Clostridium* ✓ Enhanced texture ✓ High panelists’ appreciation	De Angelis et al., 2015
Bella di Cerignola	Spanish style Brining conditions: 8 and 10% (*w*/*v*) NaCl added with glucose (0.5% *w*/*v*)	Mix of three autochthonous *L. plantarum* (c10, c16, and c19)	✓ Higher pH reduction ✓ Controlled the growth of yeasts ✓ Higher production of D- and L-lactic acids	Perricone et al., 2010
Manzanilla	Spanish style Brining conditions: 11% (*w*/*v*)	*L. pentosus* TOMC LAB2 *L. pentosus* TOMC LAB4	✓ Higher acidification ✓ Biofilm formation on olive epidermis	Rodriguez-Gomez et al., 2014
Manzanilla	Spanish-style Brining conditions: 12% (*w*/*v*)	*L. pentosus* LP99	✓ Higher pH reduction ✓ High level of 4-ethyl phenol	de Castro et al., 2019
Manzanilla	Natural style Brining conditions: 11% (*w*/*v*)	Commercial baker’s yeast (*S. cerevisiae*) *S. cerevisiae* LI-180-7 DSMZ27800	✓ Higher levels of hydroxytyrosol ✓ Higher production of lactic, acetic, malic, and tartaric acids ✓ Lower levels of cadaverine and putrescine	Tufariello et al., 2019
Manzanilla	Spanish style Brining conditions: 12.0% (*w*/*v*) NaCl, 0.13% CaCl_2_, and 0.08% HCl	Mix culture (*W. anomalus* Y12 followed by *L. pentosus* LPG1, *L. pentosus* Lp13, and *L. plantarum* Lpl15	✓ Higher pH reduction ✓ Higher AI-2 activity at the ninth day ✓ Biofilm formation	Benítez-Cabello et al., 2020a
Nocellara del Belice	Spanish style Brining conditions: 9% (*w*/*v*)	*L. pentosus* OM13	✓ Higher pH reduction ✓ Enhanced sensorial attributes	Aponte et al., 2012
Nocellara Etnea	Natural style Brining conditions: 8% (*w*/*v*)	*L. plantarum* UT2.1 + *L. paracasei* N24 + *L. pentosus* TH969 *L. plantarum* UT2.1 *L. paracasei* N24 + *L. pentosus* TH969 *L. plantarum* UT2.1 + *L. pentosus* TH969 *L. plantarum* UT2.1 + *L. paracasei* N24 *L. pentosus* TH969	✓ Higher pH reduction ✓ Enterobacteriaceae elimination ✓ High total phenolic content	Randazzo et al., 2017
Nocellara Etnea	Sicilian style Brining conditions: 5 and 8% (*w*/*v*)	Sequential inoculation of *L. plantarum* F3.3 followed by probiotic *L. paracasei* N24	✓ Higher acidification ✓ Higher debittering rate ✓ Higher polyphenol content ✓ Enterobacteriaceae elimination ✓ Higher production of phenylethyl alcohol and methyl 2-methylbutanoate ✓ Lower production of ethanol and isoamyl-alcohol ✓ Acceptance by sensory panel	Pino et al., 2019
Nocellara Etnea	Sicilian style Brining conditions: 4, 5, 6, and 8% (*w*/*v*)	Sequential inoculation of *L. plantarum* UT2.1 followed by probiotic *L. paracasei* N24	✓ Reduction of yeasts and enterobacteria ✓ Higher production of esters ✓ Highest overall acceptability (5% *w*/*v*)	Pino et al., 2018
Tonda di Cagliari	Natural style Brining conditions: 7% (*w*/*v*) Temperature: 27°C, until a steady-state pH was reached. Then, temperature was set to 24°C	*L. plantarum* (SSL) Undefined mixed culture of *L. pentosus* strains (SIE)	✓ Higher pH reduction ✓ Enterobacteriaceae elimination ✓ Higher debittering activity ✓ Higher hydroxytyrosol production ✓ Higher scavenging activity	Campus et al., 2015
Conservolea	Natural style Brining conditions: 6% (*w*/*v*)	Mix of *L. pentosus* (Lp 15, Lp 20, Lp 28, Lp 40, and Lp 48)	✓ Higher pH reduction ✓ Medium–low bitter taste ✓ Faster elimination of Enterobacteriaceae	Chranioti et al., 2018
Conservolea	Natural style Brining conditions: 8% (*w*/*v*)	*L. pentosus B281* Mix of *L. pentosus B281* and *P. membranifaciens M3A*	✓ Higher pH reduction ✓ Higher lactic and acetic acid production Lower ethanol levels ✓ Colonize olives’ surface	Grounta et al., 2016
Conservolea	Spanish style Brining conditions: 6% (*w*/*v*)	Commercial *L. pentosus* Wild-strain *L. plantarum*	✓ Faster elimination of Enterobacteriaceae ✓ Higher pH reduction ✓ Higher production of lactic and acetic acids ✓ Lower ethanol and acetaldehyde production	Panagou and Tassou, 2006
Conservolea	Natural style Brining conditions: 8% (*w*/*v*)	Sequential inoculation with *D. hansenii* A 15–44 followed by *L. plantarum* A 135–5	✓ Higher pH reduction ✓ Higher levels of esters and alcohols ✓ Higher levels in hydrocarbon content Higher overall acceptability	Chytiri et al., 2020
Halkidiki	Spanish style Heat shock (80°C for 10 min) Brining conditions: 8 and 10% (*w*/*v*)	*L. pentosus B281* *L. plantarum B282* Mix of *L. pentosus B281* and *L. plantarum B282*	✓ Higher acceptance ✓ Faster elimination of Enterobacteriaceae ✓ Faster increase of titratable acidity ✓ Higher production of lactic, acetic, and succinic acids ✓ Higher overall acceptability	Argyri et al., 2014
Halkidiki	Spanish style Brining conditions: 8 and 10% (*w*/*v*)	*L. pentosus B281* *L. plantarum B282* Mix of *L. pentosus B281* and *L. plantarum B282*	✓ Colonize olive’s surface ✓ Higher pH reduction ✓ Higher production of lactic acid ✓ Higher production of propanol and methyl ✓ Ethyl esters	Blana et al., 2014
Halkidiki	Natural style Brining conditions: o 2.3% (*w*/*v*) NaCl, 32.3 mM Ca-acetate, and 33.9 mM Ca-lactate o 4% NaCl, pH 5.0	*L. plantarum* Lp 15 *L. plantarum* Lp 20 *L. plantarum* Lp 28 *L. plantarum* Lp 40 *L. plantarum* Lp 48	✓ Increase of hydroxytyrosol and tyrosol	Kaltsa et al., 2015
Kalamata	Natural style Brining conditions: o 2.3% (*w*/*v*) NaCl, 32.3 mM Ca-acetate, and 33.9 mM Ca-lactate o 4% NaCl, pH 5.0	*L. plantarum* Lp 15 *L. plantarum* Lp 20 *L. plantarum* Lp 28 *L. plantarum* Lp 40 *L. plantarum* Lp 48	✓ Increase of hydroxytyrosol and tyrosol production	Kaltsa et al., 2015
Kalamata	Natural style Brining conditions: 11% (*w*/*v*)	Commercial preparation of baker’s yeast (*S. cerevisiae*) *S. cerevisiae* LI-180-7 DSMZ27800	✓ Higher levels of hydroxytyrosol ✓ Higher production of lactic, acetic, malic, and tartaric acids ✓ Lower levels of cadaverine and putrescine	Tufariello et al., 2019
Kalamata	Natural style Brining conditions: 5% (*w*/*v*)	*L. mesenteroides* Lm139 *L. pentosus* DSM 16366	✓ Faster brine acidification ✓ Higher production of lactic acid ✓ Faster elimination of Enterobacteriaceae	Papadelli et al., 2015
Cyprus	Natural style Brining conditions: 7 and 10% (*w*/*v*) acidified with citric acid	Commercial *L. plantarum* (Vege-Start 60)	✓ Higher acceptance ✓ Faster elimination of Enterobacteriaceae ✓ Faster pH reduction ✓ Higher production of lactic acid ✓ Faster oleuropein degradation	Anagnostopoulos et al., 2020a
Picual	Natural style Brining conditions: 7 and 10% (*w*/*v*) acidified with citric acid	Commercial *L. plantarum* (Vege-Start 60)	✓ Higher acceptance ✓ Faster elimination of Enterobacteriaceae ✓ Faster pH reduction ✓ Higher production of lactic acid ✓ Higher levels of hydroxytyrosol ✓ Higher antioxidant capacity	Anagnostopoulos et al., 2020b
Picual	Natural style Brining conditions: 11% (*w*/*v*)	Commercial baker’s yeast (*S. cerevisiae*) *S. cerevisiae* LI-180-7 DSMZ27800	✓ Higher levels of hydroxytyrosol ✓ Higher production of lactic, acetic, malic, and tartaric acids ✓ Lower levels of cadaverine and putrescine	Tufariello et al., 2019
Taggiasca	Natural style Brining conditions: 12% NaCl acidified with citric acid	*C. diddensiae* 2011 *C. adriatica* 1985 *W. anomalus* 1960	✓ Faster pH reduction ✓ Lower bitter taste	Ciafardini and Zullo, 2019

*^1^Main findings and advantages compared to the conventional/spontaneous fermentation.*

Similarly, although there is a huge number of studies providing the benefits of using yeast starters, either alone (Ciafardini and Zullo, 2019; Schaide et al., 2019; Tufariello et al., 2019) or in combination with LAB (Pistarino et al., 2013; De Angelis et al., 2015; Tufariello et al., 2015; Benítez-Cabello et al., 2019a,2020a; Chytiri et al., 2020; Garrido-Fernández et al., 2021), in olive fermentation, the use of yeasts as starters is still at research level (Table 1). The latter strategy seems to be the most promising one, since as previously mentioned, it has been proven that the presence of yeasts favors the growth of LAB population, by (a) degrading complex compounds (e.g., oleuropein and phenolics), which act as inhibitors to LAB, or (b) producing several elements, which are crucial for LAB development (e.g., vitamins and purines) (Arroyo-López et al., 2012b; Anagnostopoulos et al., 2017). Indeed, De Angelis et al. (2015) reported very promising findings by using LAB strains in combination with an autochthonous *W. anomalus* strain especially regarding the acceleration of the fermentation of Bella di Cerignola table olives. Furthermore, the use of *P. membranifaciens* in combination with a *L. pentosus* strain was successfully applied in the fermentation of Conservolea black olives, leading to the production of a final product with an improved sensory profile (Grounta et al., 2016). Additionally, Tufariello et al. (2015) applied a sequential inoculation strategy of four yeast starters followed by four LAB strains in four different olive cultivars, respectively. The results indicated a shortened time of fermentation process and a significant improvement of the sensorial attributes of the final product. All the aforementioned findings confirm the claim of the synergistic symbiosis between LAB and yeasts, highlighting the significant contribution of yeasts in a mix culture to (a) ensure the survival and enhance the growth of LAB and (b) provide several benefits in the final product, regarding organoleptic features such as flavor and aroma. Finally, Ciafardini et al. (2021) have recently introduced a novel and low-cost strategy to produce Taggiasca black table olives. More specifically, the authors used an already fermented brine (containing mainly *Pichia manshurica* and *S. cerevisiae* strains) as a starter to drive the fermentation process. The results indicated several positive effects compared to the conventional process, especially on the debittering course and the reduction of fermentation time (shortened by about 3 months). However, the authors noted the importance of choosing brine starters after applying an in-depth study to those brines regarding both physicochemical and microbiological features, to reduce the risk of potential deterioration on both fermentation course as well as on the sensorial attributes of the final product. Such kind of work requires further evaluation in the upcoming years, since it is not only a novel and very promising strategy for table olive production but it also touches on the wastes’ sustainable management.

As mentioned above, despite the readily available literature, in which the use of starter is strongly recommended to be established, the industry has yet to adapt this concept, remaining on conventional and/or traditional and/or local production approach. This may be the outcome of the limited cooperation between the scientific and industrial communities, as well as the producers’ knowledge gap regarding the concept of starter cultures and their potential benefits. To overcome this problem and proceed to the next level, a collaborative partnership between scientists, producers, and any other stakeholders is the most promising strategy. Furthermore, the involvement of many types of consumers supporting healthy nutrition (e.g., vegetarians and vegans), in a synergistic action with the industry and their awareness and continuing briefing regarding the concept of potential probiotic starter cultures, is a strategy of great importance that deserves major attention in the next few years.

## The Contributions of High-Throughput Sequencing Approach in the Olive Fermentation Field: Current Applications and Future Perspectives

In the last decade, the development of novel culture-independent methods and especially HTS technology provided new insights in the study of food-associated microbiota, replacing and/or supplementing the conventional methods (both culture dependent and culture independent). The important contribution of such a modernized approach is reflected by the fact that several microbial groups, previously not detected, have surfaced (Cocolin et al., 2013b). Given that, it is crucial to point out the need of re-reviewing our thoughts and knowledge regarding microbial presence and ecology in a food sample, opening a new frontier in the field of food microbiology, the so-called foodomics, which is a variety of different related omics angles such as genomics, transcriptomics, proteomics, and metabolomics (Balkir et al., 2021). Nowadays, foodomics studies are evaluated at the metagenome level, and thus, the meta-omics approach has arisen. Like many other foods, the study of table olive entered into this era, the application of which makes it feasible to reveal the real picture on microbial ecology during olive processing (Vaccalluzzo et al., 2020b).

According to the literature, there have been several studies already applied on table olive fermentation, in terms of metabarcoding by using several HTS technologies such as Illumina and Roche (Cocolin et al., 2013a; De Angelis et al., 2015; Arroyo-López et al., 2016; Randazzo et al., 2017; Zinno et al., 2017; de Castro et al., 2018; Medina et al., 2018; Benítez-Cabello et al., 2019b,2020a; Anagnostopoulos et al., 2020b; Kazou et al., 2020; Penland et al., 2020; Demirci et al., 2021; López-García et al., 2021; Michailidou et al., 2021). Table 2 summarizes the main microbial groups detected by studies so far through metataxonomic approach, in different olive varieties and processes. For instance, Medina et al. (2018) evaluated the microbiota present during processing of Spanish olives darkened by oxidation, highlighting the dominance of *Acetobacter*, *Lactiplantibacillus*, and *Oenococcus*, noting in parallel the presence of some bacteria (*Pseudoalteromonas*, *Alteromonas*, *Marinomonas*, and *Oenococcus*) that have never been previously reported in table olives. Furthermore, the dominance of *Lactiplantibacillus* starters was determined in naturally fermented Bella di Cerignola (De Angelis et al., 2015), Picual (Anagnostopoulos et al., 2020b), Spanish-style fermented Manzanilla (Benítez-Cabello et al., 2020a), and the Sicilian Nocellara Etnea (Randazzo et al., 2017), since this genus was the most abundant at the end of the process. Rodríguez-Gómez et al. (2017) revealed the dominance of *Lactiplantibacillus*, followed by *Pediococcus* and *Celerinatantimonas*, in natural green, heat-shocked Aloreña de Málaga olives. In another study, Cocolin et al. (2013a) examined the potential effects of NaOH on the microbiota of Etnea olives. The results indicated a completely different bacterial profile between the control and treated samples, in which the dominant genera were *Lactiplantibacillus* and Enterobacteria, respectively. Furthermore, Arroyo-López et al. (2016) highlighted the dominance of *Pichia* and *Zygotorulaspora* at the end of the process of naturally fermented Aloreña de Málaga. Moreover, Zinno et al. (2017) noted that the dominant microbiota of Nocellara del Belice table olives fermented by partial substitution of NaCl by KCl at concentrations of 25, 50, and 75% was *Lactiplantibacillus*, Enterobacteriaceae, and *Lactiplantibacillus* followed by *Pediococcus*, respectively. Benítez-Cabello et al. (2020b) studied the bacterial diversity in non-thermally treated commercial table olive biofilms. The authors reported that the biofilm consortium was dominated by *Lactiplantibacillus*, followed by *Pediococcus*, while the presence of other bacterial groups (e.g., *Celerinatantimonas*, *Leuconostoc*, *Marinilactibacillus*, and Enterobacteriaceae) was more limited. Those findings are in line with a recent work by López-García et al. (2021), where the microbiota of packed Aloreña de Málaga olives was evaluated. In the same study, the presence of yeasts was also studied, indicating the dominance of *Citeromyces* followed by *Candida* and *Penicillium*. Furthermore, *Citeromyces* and *Pichia* were reported to play a key role during fermentation of Nyons Black Olive (Penland et al., 2020). Finally, de Castro et al. (2018) studied the bacterial and metabolite profile of spoiled green olives, indicating the high abundance of *Cardiobacteriaceae* and *Ruminococcus*. It is crucial to mention that Pearson’s correlation revealed the link of *Ruminococcus* with the production of both propionic and butyric acids.

**TABLE 2:** Detection of the main microbiota present in different fermentation types of several table olive cultivars, as revealed by high-throughput sequencing approach.

Table olive	Fermentation style	HTS method/targeted region	Detected microbiota	Dominant microbiota	References
Aloreña de Málaga	Natural/heat-shocked/15.8% NaCl (*w*/*v*)	Illumina/V3–V4 region of 16S rRNA gene	*Lactiplantibacillus* *Pediococcus* *Marinilactibacillus*	*Lactiplantibacillus*	Rodríguez-Gómez et al., 2017
Hojiblanca Manzanilla	Spanish style	Illumina/V2–V3 region of 16S rRNA gene	*Lactiplantibacillus* *Oenococcus* *Enterococcus* *Lactococcus* *Weissella* *Leuconostoc* *Streptococcus* Enterobacteriaceae *Vibrio*	*Lactobacillus* *Oenococcus*	Medina et al., 2018
Nocellara del Belice	Sivigliano (control, 25, 50, and 75% replacement with KCl)	Illumina/V3–V4 region of 16S rRNA gene	*Lactobacillales* *Lactiplantibacillus* *Pediococcus* *Lactococcus*	*Lactobacillales* *Lactiplantibacillus*	Zinno et al., 2017
Nocellara del Belice	Castelvetrano (control, 25, 50, and 75% replacement with KCl)	Illumina/V3–V4 region of 16S rRNA gene	*Lactiplantibacillus* *Pediococcus* *Halomonas* *Marinilactobacillus* *Natronobacillus* *Alkalibacterium*	*Lactiplantibacillus* *Marinilactobacillus* (control and 75% KCL)	Zinno et al., 2017
Picual	Natural style/7 and 10% NaCl (*w*/*v*)	Illumina/V3–V4 region of 16S rRNA gene	*Lactiplantibacillus* *Pediococcus*	*Lactiplantibacillus*	Anagnostopoulos et al., 2020b
Nocellara Etnea	Natural style/8% NaCl (*w*/*v*)	Tag-encoded FLX amplicon pyrosequencing of both DNA and RNA	*Chromohalobacter* *Halomonas* *Marinilactibacillus* *Flavobacterium* *Lactiplantibacillus*	*Halomonas* *Marinilactibacillus*	Cocolin et al., 2013a
Nocellara Etnea	Spanish style/treated with NaOH (1% *w*/*v*)	Tag-encoded FLX amplicon pyrosequencing of both DNA and RNA	*Lactiplantibacillus* *Marinilactibacillus*	*Lactiplantibacillus*	Cocolin et al., 2013a
Aloreña de Málaga	Packed/55 g/l NaCl, 0.8 g/l lactic acid, 1 g/l ascorbic acid, 3 g/l citric acid, 2 g/l potassium sorbate, and 1 g/l sodium benzoate	Illumina/V3–V4 region of 16S rRNA gene for bacteria/ITS1–ITS2 region for fungi	Carnobacteriaceae Lactobacillaceae Celerinatantimonadaceae Oceanospirillales Cardiobacteriales Enterobaterales Aspergillaceae Debaryomycetaceae Phaffomycetaceae Citeromyces	Carnobacteriaceae Lactobacillaceae Celerinatantimonadaceae Aspergillaceae Debaryomycetaceae Phaffomycetaceae	López-García et al., 2021
Commercial table olive from different supermarkets worldwide	–^1^	Illumina/V3–V4 region of 16S rRNA gene	*Lactiplantibacillus* *Pediococcus* *Celerinatantimonas* *Leuconostoc* *Alkalibacterium* *Pseudomonas* *Marinilactibacillus* *Weissella* Enterobacteriaceae	*Lactiplantibacillus*	Benítez-Cabello et al., 2020b
–^1^	Diverse aromatic herbs (thymus, fennel, and oregano) Fermented dressing materials (sliced red pepper, pepper paste, and garlic) Salts (marine and spring salt)	Illumina/V3–V4 region of 16S rRNA gene	*Pseudomonas* Enterobacteriaceae *Lactiplantibacillus* Weissella *Pediococcus* *Salinibacter* *Sphingomonas*	*Pseudomonas* Enterobacteriaceae (aromatic herbs) *Lactiplantibacillus* *Weissella* *Pediococcus* (fermented dressing samples) *Salinibacter* (salts)	Benítez-Cabello et al., 2019b
Kalamata	Packed in multi-layered pouches under modified atmosphere (30% CO_2_–70% N_2_)	Illumina/V3–V4 region of 16S rRNA gene for bacteria/V7–V8 region of 18S rRNA gene for fungi	*Lactiplantibacillus* *Pediococcus* *Curvibacter* *Sphingomonas* *Pannonibacter* *Phenylobacterium* *Enhydrobacter* *Alishewanella* *Pichia* *Brettanomyces* *Issatchenkia* *Cladosporium*	*Lactiplantibacillus* *Brettanomyces*	Michailidou et al., 2021
Turkish, green cracked	Natural style/8–12% NaCl (*w*/*v*)	–^1^	*Saccharomyces* Tetrapisispora Naumovozyme Candida Debaryomyces Millerozyma Spathaspora Wickerhamomyces Komagataella Gammaproteobacteria Bacilli Enterobacteriaceae Lactobacillales Aerococcus Streptococcaceae	Saccharomycetales Agaricomycetes Gammaproteobacteria	Demirci et al., 2021
Nyons	Natural style/10% NaCl (*w*/*v*)	Illumina/V3–V4 region of 16S rRNA gene for bacteria/ITS2 region for fungi	*Citeromyces* *Wickerhamomyces* *Zygotorulaspora* *Candida* *Pichia* *Celerinatantimonas* *Photobacterium* *Marinobacterium* *Bacillus* *Leuconostoc* *Lactococcus* *Lactiplantibacillus*	*Citeromyces* *Wickerhamomyces* *Zygotorulaspora* *Candida* *Pichia* *Celerinatantimonas*	Penland et al., 2020

*^1^Not provided.*

Metataxonomic studies have enriched in-depth our knowledge on table olive microbiota, revealing the presence of several microbial groups involved during fermentation, which could not be detected using conventional methods. However, this technique does not provide any information about microbial functionality, giving just a snapshot of microbial presence (Cocolin et al., 2018). Even though there are some studies in which metataxonomic data are statistically combined with other parameters, such as VOCs (Randazzo et al., 2017; de Castro et al., 2018), De Filippis et al. (2017) highlighted the probability of a potential statistically based linkage between a microbial group with a VOC group, with no scientific meaning. Thus, further and more advanced research is needed to be applied in order to unveil the microbial functionality during olive fermentation. The couple of meta-omics (multi-omics), combining the study approach of metataxonomics with metagenomics, is the key to achieve this effort (Cocolin et al., 2018). The application of multi-omics during olive fermentation could answer crucial aspects that we were unable to address until nowadays, giving rise in parallel to new critical questions for stepping forward to new insights (De Filippis et al., 2017). Furthermore, multi-omics may contribute to the development of a network, which, in turn, may lead to unveil the complex system of foods’ fermentation and develop novel strategies to better control the fermentation process, resulting in an invaluable impact on the final product’s quality. To our knowledge, in the field of olive fermentation, no such type of study has been applied so far. The exploitation of dominant microbiota, gene, and protein expression, as well as the identification of the real effect of those mechanisms on the organoleptic features of the final product, is one of the most needed works that has to be applied in the olive’s field, as has already happened in the study of other fermented foods. As a representative example, Zhao et al. (2019) provided an advanced study on the microbial ecology of naturally fermented Chinese puerh tea by combining metabarcoding, metaproteomics, and metabolomics. The results revealed that a part of microbiota produced CAZymes to degrade plant or fungal polysaccharides for their growth and reproduction, as well as several enzymes involved in the hydrolysis, oxidization, modification, or degradation of phenolic compounds. Additionally, Xie et al. (2019) unveiled several microbial assortments responsible for the production of key enzymes in bean sauce mash. Similar works should be applied to the unexplored field of table olive fermentation, to obtain useful information about important functionalities taking place during processing. Finally, according to Vaccalluzzo et al. (2020b), this kind of study approach could potentially lead to the detection of novel biomarkers, which in turn could be used to develop novel and intelligent strategies, to guarantee the safety, reproductivity, and succession of fermentation and, thus, produce a stable final product with potential added value.

Another frontier of great interest is olives’ typicity. In the last 20 years, the concept of “microbial terroir” has been introduced, in the altar of saying that specific environments may maintain specific microbial distributions (Martiny et al., 2006). In olives’ field, the idea of biogeographic fingerprint was recently introduced by Lucena-Padrós and Ruiz-Barba (2019). The authors studied the microbial distribution of Spanish-style green olives from different areas of Seville. However, the study was applied using a culture-dependent approach. Nowadays, the scientific community should take advantage of the rapid development of HTS technology attempting to establish potential terroir, based on microbiota present in olive samples from different geographical regions (Bokulich et al., 2014). Like other naturally fermented products (e.g., wine), olive is a clear natural one, meaning that the majority of microbial communities that lead the fermentation are coming from the olives’ microenvironment. In this sense, connecting olives’ microbiota with a specific region could lead to the promotion of several cultivars as potential PDO/PGI, increasing in parallel their added value. The latter could open new insights in the field of olive industry and production, including high recognition and economic benefits for the producing countries.

There have already been applied studies aiming to examine the potential geographical impact on olives using HTS approach in combination with other parameters, such as physicochemical ones. For instance, the effect of geographical origin on the microbiota of two well-known olive cultivars (cv. Konservolia and cv. Halkidiki) was recently assessed by Argyri et al. (2020). The authors highlighted the significant contribution of partial least squares-discriminant analysis (PLS-DA) to combine metataxonomic, microbiological, physicochemical, and organoleptic data, indicating satisfactory discrimination among table olives’ microbial diversity from different geographical regions, while the pH, total viable counts (TVCs), and LAB plate counting represented the most discriminative parameters. Furthermore, Kazou et al. (2020) assessed the potential microbial fingerprint in cv. Kalamata olives from two different Greek regions (Aitoloakarnania and Messinia/Lakonia) by applying metataxonomic analysis. The authors highlighted satisfactory discrimination between the two regions, especially by yeast metataxonomic analysis in the brine samples. Both studies reveal the claim that specific microbial groups are associated with specific geographic areas. However, as it is observed, the available literature regarding this kind of research is very limited, and thus, further similar studies are needed to enhance the hypothesis of microbial terroir, opening a new interesting and challenging insight in the field of table olives.

## Conclusion

To sum up, the olive sector will face several challenges in the near future, which could be divided into six main parts: (a) the full understanding of table olive microbial ecology by the use of multi-omics; (b) the selection of the most promising microbes, regarding their technological and potential probiotic properties; (c) fermentation control *via* the application of the selected starter culture (LAB, yeast, or mixed) and high cooperation between scientists, producers, and consumers for its official establishment; (d) the standardization of the inoculum production method and the reduction of NaCl content; (e) the determination of potential functional properties of the starter-driven olives *via* clinical studies; and (f) a potential microbiota-based olives’ typicity determination using HTS approach. More specifically, the application of starter cultures, especially the autochthonous ones, in combination with the use of reducing NaCl levels during fermentation represents the most promising biotechnological innovation of this field. Furthermore, the use of HTS in an attempt to highlight potential “key microbial groups” that may be associated with a specific basin, as well as the combination of meta-omics to understand the functions responsible for specific and/or unique sensorial traits of the final product, is the biggest challenge of the current decade. This kind of work is now more essential than ever before, to establish and link the concept of microbial terroir with olives’ organoleptic characteristics, as an attempt to highlight table olives with microbiota-based PDO/PGI typicity, opening the new era in olives’ frontier. To conclude, we are standing at a cross point where the present is meeting the future. It is expected that the majority, if not all, of the aforementioned challenges will be achieved in the coming years, and thus, the running and/or the next decade could potentially become “the golden decade” of table olives.

## Author Contributions

DA and DT wrote and reviewed the manuscript. Both authors contributed to the article and approved the submitted version.

## Conflict of Interest

The authors declare that the research was conducted in the absence of any commercial or financial relationships that could be construed as a potential conflict of interest.

## Publisher’s Note

All claims expressed in this article are solely those of the authors and do not necessarily represent those of their affiliated organizations, or those of the publisher, the editors and the reviewers. Any product that may be evaluated in this article, or claim that may be made by its manufacturer, is not guaranteed or endorsed by the publisher.
